# Impact of individual and treatment characteristics on wearable sensor-based digital biomarkers of opioid use

**DOI:** 10.1038/s41746-022-00664-z

**Published:** 2022-08-22

**Authors:** Brittany P. Chapman, Bhanu Teja Gullapalli, Tauhidur Rahman, David Smelson, Edward W. Boyer, Stephanie Carreiro

**Affiliations:** 1 Department of Emergency Medicine, Division of Medical Toxicology, University of Massachusetts Chan Medical School, Worcester, MA USA; 2 College of Information and Computer Sciences, University of Massachusetts Amherst, Amherst, MA USA; 3 Department of Psychiatry, Division of Addiction Psychiatry, University of Massachusetts Chan Medical School, Worcester, MA USA; 4 Department of Emergency Medicine, The Ohio State University, Columbus, OH USA

**Keywords:** Diagnostic markers, Translational research

## Abstract

Opioid use disorder is one of the most pressing public health problems of our time. Mobile health tools, including wearable sensors, have great potential in this space, but have been underutilized. Of specific interest are digital biomarkers, or end-user generated physiologic or behavioral measurements that correlate with health or pathology. The current manuscript describes a longitudinal, observational study of adult patients receiving opioid analgesics for acute painful conditions. Participants in the study are monitored with a wrist-worn E4 sensor, during which time physiologic parameters (heart rate/variability, electrodermal activity, skin temperature, and accelerometry) are collected continuously. Opioid use events are recorded via electronic medical record and self-report. Three-hundred thirty-nine discreet dose opioid events from 36 participant are analyzed among 2070 h of sensor data. Fifty-one features are extracted from the data and initially compared pre- and post-opioid administration, and subsequently are used to generate machine learning models. Model performance is compared based on individual and treatment characteristics. The best performing machine learning model to detect opioid administration is a Channel-Temporal Attention-Temporal Convolutional Network (CTA-TCN) model using raw data from the wearable sensor. History of intravenous drug use is associated with better model performance, while middle age, and co-administration of non-narcotic analgesia or sedative drugs are associated with worse model performance. These characteristics may be candidate input features for future opioid detection model iterations. Once mature, this technology could provide clinicians with actionable data on opioid use patterns in real-world settings, and predictive analytics for early identification of opioid use disorder risk.

## Introduction

Opioid use disorder (OUD) is one of the most pressing public health problems of our time, with staggering morbidity, mortality, social impact, and economic costs. In 2021, roughly twelve lives were lost in the United States every 60 min to an overdose death, and more than half of these deaths were linked to opioids^1^. Overdose-related death however, is only one of the many devastating consequences of OUD; the associated morbidity also has significant physical, social, and financial tolls^2–5^. Prescription opioids play a critical role in the opioid crisis as they increase exposure and availability in the general population. Increased opioid prescribing has been clearly linked to problematic opioid use, and prescription opioids are often the first source of exposure for individuals who go on to develop OUD. This makes prescription opioids a compelling target for prevention and risk mitigation strategies.

The rapidly expanding field of mobile health (mHealth) could provide unique advantages to prescription opioid monitoring. Of specific interest are digital biomarkers, or end-user generated physiologic or behavioral measurements that correlate with events of interest, health, or pathology^6–8^. Potential benefits of mHealth devices include portability, low cost, and ease of use that make them particularly attractive solutions. With regards to clinical applications within the opioid use space, a robust mHealth ecosystem could provide support for healthcare providers across the spectrum of care. Automated, objective detection of opioid ingestion could give providers valuable data on the the quantity and patterns of opioid use, and model how the individuals’ physiologic response to opioids changes over time. These models could be correlated with clinical outcomes, and used to predict risk of OUD using individualized adaptive learning strategies. Beyond monitoring, digital biomarkers of opioid use could be used to trigger just-in-time (and just-in-space) adaptive interventions to mitigate the risk of opioid misuse or OUD. In individuals with OUD, opioid use detection could be leveraged as a harm reduction strategy by adapting models for opioid overdose detection. And finally for those in recovery, mHealth tools could be leveraged to augment treatment with the partial opioid agonist buprenorphine. However, first an accurate empirical model for the detection of opioid use events must be established, and the baseline algorithm needs to be optimized to understand what patient and treatment level factors impact digital biomarkers of opioid use.

Preliminary work has demonstrated that physiologic changes are evident in wearable sensor data surrounding opioid use and that there are qualitative differences on those sensor-based biomarkers depending on opioid exposure history (i.e., previously opioid-naive individuals versus those with a history of chronic use). Individuals with chronic opioid use display physiologic changes consistent with withdrawal symptoms immediately prior to an opioid administration^9,10^, which is intuitive given our knowledge of opioid dependence over time. However, other factors are expected to play an important role in an individual’s response to opioids, and therefore in our ability to detect them. For example, sex-based differences in overall kinematics^11,12^ and heart rate variability^13^ are expected to impact accelerometry and photoplethysmography (PPG) measurements, respectively. Specific to opioid effect, we expect to see sex-based differences on sedation, locomotion, and analgesia^14–16^. Age is an individual attribute expected to impact parameters such as heart rate variability (HRV)^17^ and locomotion^18^. Concomitantly administered medications and medical interventions also create potential confounders in our ability to continuously measure opioid physiology. Sympatholytics (i.e., beta-adrenergic antagonists) and sedatives are expected to blunt increases in heart rate and electrodermal activity (EDA), while stimulants (i.e., amphetamines, nicotine) are expected to exaggerate changes in these parameters.

Similarly, understanding the contribution and importance of each sensor feature in our ability to detect digital biomarkers of opioid use is crucial to optimizing a sensor array. Each sensor consumes energy, creates computational cost through its data processing needs, and increases the expense of the device. Identifying the optimal data streams needed (and perhaps more importantly, identifying those that are not needed) will ultimately lower cost, minimize computational complexity, save battery life, and allow for the use of more compact and aesthetically appealing sensors.

To address these knowledge gaps, we collected a longitudinal dataset from hospitalized patients receiving repeated doses of opioid analgesics to achieve the following aims: (a) Characterize wearable sensor-based feature changes that occur with repeated opioid administration; (b) Train and optimize an updated machine learning model on these data to detect opioid use events; and, (c) Explore which individual and treatment factors are associated with model performance including sex, substance use history, medical history, and concomitantly administered medications.

## Results

### Sample participant characteristics and opioid administration patterns

Thirty-six participants were enrolled in this study. The sample was 42% female and 83% Caucasian. Additional participant characteristics are outlined in Table 1. Forty-two percent of participants were classified as individuals who use opioids chronically, 47% had received at least one opioid prescription in the last year, and 81% reported using at least one substance at baseline (inclusive of tobacco and/or alcohol (Table 2). Mean duration of study participation (hospitalization) was 3.8 days (±3.3, range 1–14).

**Table 1:** Participant demographics.

	Overall
	(*N* = 36)
Age (in years)	
Mean (SD)	50.6 (14.8)
Median [Min, Max]	48.5 [22.0, 79.0]
Sex	
Male	21 (58.3%)
Female	15 (41.7%)
Race	
American Indian or Alaska Native	2 (5.6%)
Black or African American	2 (5.6%)
White	30 (83.3%)
Hispanic/Latinx	2 (5.6%)
Dominant Hand	
Left	4 (11.1%)
Right	30 (83.3%)
Body Mass Index	
Mean (SD)	28.5 (6.55)
Median [Min, Max]	28.0 [16.6, 42.6]
Chronic Pain History	16 (44.4%)
Psychaitric History	24 (66.7%)

**Table 2:** Participant substance use history.

	Overall
	(*N* = 36)
Opioid Use Class	
Naive	14 (38.9%)
Occasional	7 (19.4%)
Chronic	15 (41.7%)
Substance Use, Current	29 (80.6%)
Substance Use Type, Current	
Tobacco	4 (11.1%)
EtOH	7 (19.4%)
Cannabis	3 (8.3%)
Heroin	0 (0%)
Other Opioids	2 (5.6%)
Substance Use Disorder Diagnosis, Lifetime	24 (66.7%)
Tobacco Use, Lifetime	29 (80.6%)
Alcohol Use, Lifetime	
Social	12 (33.3%)
Moderate/binge	6 (16.7%)
Heavy	11 (30.6%)
IVDU, Lifetime	4 (11.1%)

Five of the participants had no recorded opioids administered during the study period, despite having been prescribed them. A total of 2070 h of sensor data were obtained, and 339 discreet dose intravenous opioid administrations were captured. One hundred thirty-four hours (6.5%) of sensor data were removed due to non-physiologic values, as described in the Methods section (Machine Learning Model Development). The most common opioid administered was morphine (69% of administrations) followed by hydromorphone (31%). The mean morphine milligram equivalents (MME) per day was 64.2 (±57.0, range 0.0–240.0), and mean MME over the course of the study was 213.8 (±316.0, range 18.0–1,577.0).

### Performance of individual sensor features

**Fig. 1:**
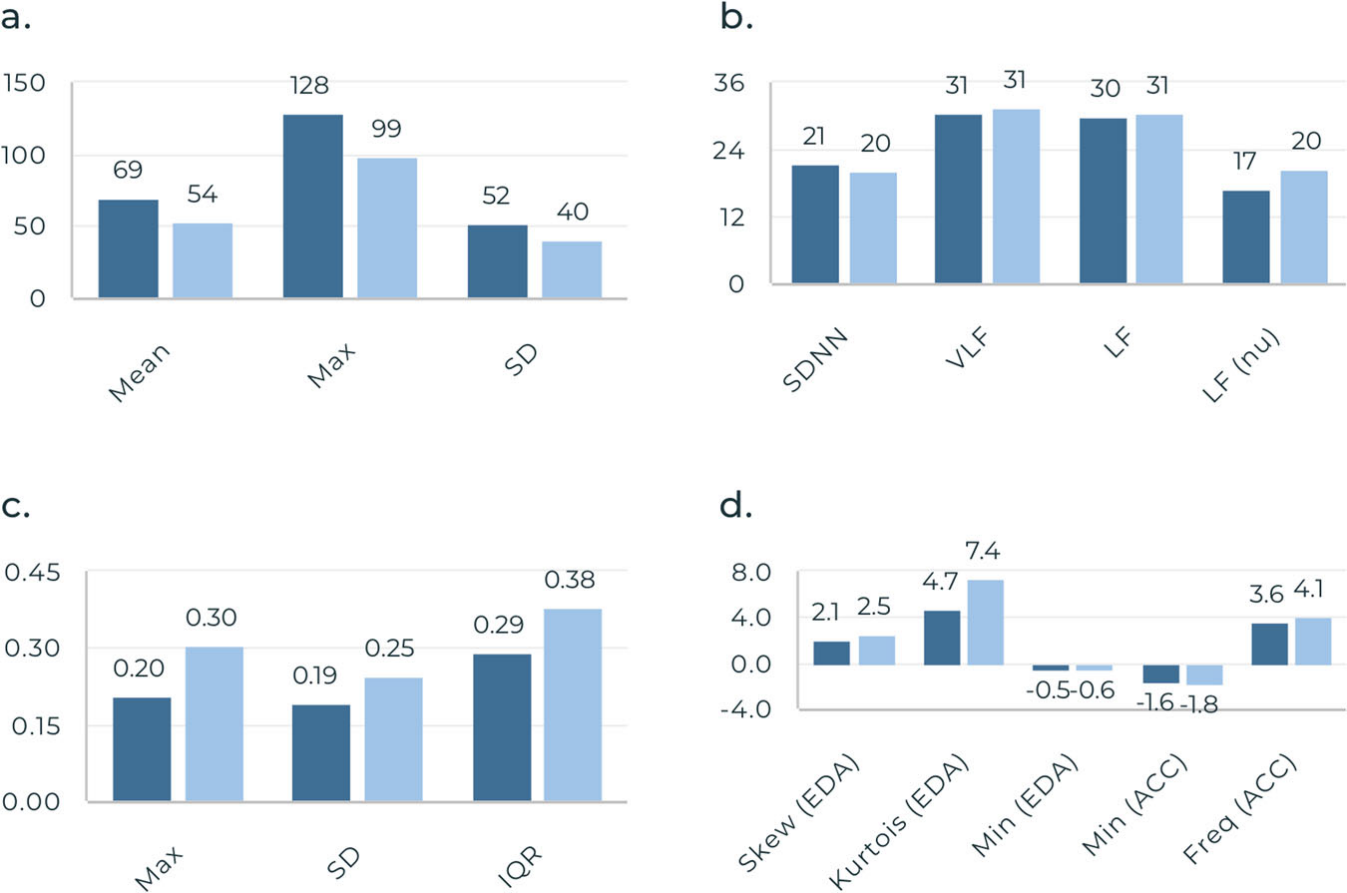
Significant features pre- to post-opioid administration. **a** HR Features, **b** IBI Features, **c** Skin Temperature Features, and **d** EDA and ACC Features. Dark blue Pre-Opioid Mean, Light Blue Post-Opioid Mean. Max Maximum, SD Standard deviation, IBI Interbeat interval, SDNN SD of NN intervals, VLF Logarithmic of absolute power of very low frequency band, LF Logarithmic of absolute power of low frequency band, LF(nu) Normalized absolute power of LF band, IQR Interquartile range, EDA Electrodermal Activity, ACC Accelerometry, Min Minimum, Freq Frequency.

Of the sensor-derived features listed in Table 3, statistically significant differences were found from pre- to post-administration in multiple domains including: a increase in accelerometer (ACC) mean frequency, increase in maximum skin temperature, increase in EDA standard deviation (SD), decrease in mean heart rate (HR), and increase in low frequency (LF) HRV (Fig. 1). A complete list of significant features and corresponding F scores are listed in the Supplementary Material (Supplementary Table 1).

**Table 3:** List of features extracted from E4 physiological sensor signals.

Domain	Sensor data stream	Feature
Time-Domain	Accelerometry	Minimum
	Electrodermal Activity	Maximum
	Skin temperature	Mean
	Heart rate	Median
		SD
		Skewness
		Kurtosis
		Interquartile range
	Interbeat interval	MeanNN
		SDNN
		RMSSD
		SDSD
		NN50
		pNN50
Frequency-Domain	Accelerometry	Dominant frequency
		Spectral entropy
		Spectral energy
		Minimum
		Maximum
		Mean
		SD
	Interbeat interval	VLF
		LF
		HF
		LF/HF ratio
		LF (nu)
		HF (nu)

*SD* Standard deviation, *SDNN* Standard deviation of NN intervals obtained from the time-window, *RMSSD* Root mean square of successive differences between heartbeats in the time-window, *SDSD* Standard deviation of differences between consecutive NN intervals, *NN50* The number of successive NN interval that differ by more than 50 ms in the time-window, *pNN50* The percentage of successive NN interval that differ by more than 50 ms in the time-window, *VLF* Logarithmic of absolute power of very low frequency band (0–0.04 hz), *LF* Logarithmic of absolute power of low frequency band, *LF(nu)* Normalized absolute power of low frequency band, *HF(nu)* Normalized absolute power of High frequency band.

### Machine learning model to detect opioid administration

**Fig. 2:**
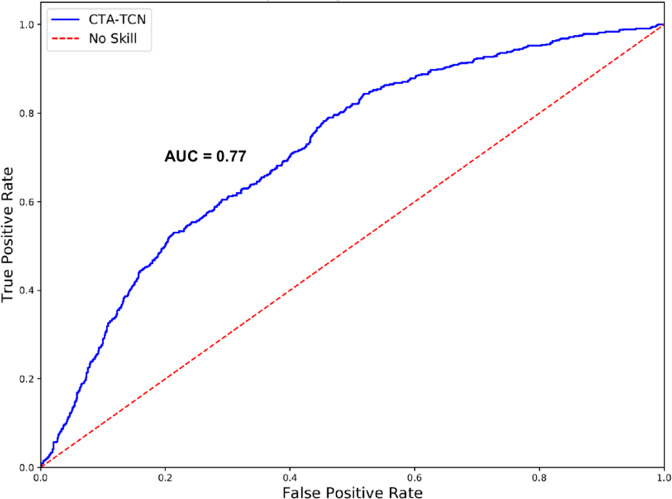
ROC curve for CTA-TCN model. CTA-TCN Channel-Temporal Attention-Temporal Convolutional Network. AUC Area Under the Curve. ROC Receiver Operator Characteristic.

After developing all of the models described in the Methods Section (Machine Learning Model Development) using both raw sensor data and the 51 calculated features described in there Methods Section (Feature Performance Analysis, a Channel-Temporal Attention-Temporal Convolutional Network (CTA-TCN) model using only raw sensor data demonstrated the best overall performance to predict opioid administration events (Table 4). A CTA-TCN model incorporates both temporal and spatial data in outcome prediction decisions. In the case of the present dataset, it allows for sequential prediction of our two outcomes of interest: first, it will predict whether or not an opioid administration has occurred (positive class), and if the positive class is predicted, then it will predict when in the data window it occurred. Model performance was optimized with a window size of 100 min, a sliding window of 20 min, and an opioid administration in the center of the window (at 50 min). The best performing model had the following metrics: model’s F1 score of 0.80 ± 0.10, specificity of 0.77 ± 0.14, sensitivity of 0.80 ± 0.17, area under the curve (AUC) of 0.77 ± 0.10, mean-absolute error (MAE) of 8.6 min ±2.4, and *R*^2^ coefficient 0.85. The receiver operating characteristic (ROC) curve for this model is presented in Fig. 2. Details and development of the CTA-TCN model architecture are out of the scope of this paper, and are described elsewhere^19^).

**Table 4:** Performance metrics for all machine learning models.

Model	F1 Score	Specificity	Sensitivity	AUC
Logistic	0.64 ± 0.13	0.65 ± 0.14	0.48 ± 0.25	0.55 ± 0.17
BiLSTM	0.70 ± 0.10	0.71 ± 0.20	0.57 ± 0.30	0.70 ± 0.14
TCN	0.73 ± 0.11	0.72 ± 0.14	0.74 ± 0.18	0.74 ± 0.11
CNN-LSTM	0.72 ± 0.11	0.65 ± 0.17	0.82 ± 0.12	0.76 ± 0.12
LSTM-FCN	0.70 ± 0.08	0.71 ± 0.16	0.69 ± 0.14	0.72 ± 0.15
CTA-TCN^a^	0.80 ± 0.10	0.77 ± 0.14	0.80 ± 0.17	0.77 ± 0.10

*AUC* Area under the curve, *BiLSTM* Bidirectional Long Short-Term Memory, *TCN* Temporal Convolutional Network, *CNN-LSTM* Convolutional Neural Network Long Short-Term Memory, *LSTM-FCN* Long Short-Term Memory with Fully Convolutional Network, *TCA-CTN* Channel-Temporal Attention-Temporal Convolutional Network.

^a^Denotes best performing model.

### Model performance stratified by individual characteristics

**Fig. 3:**
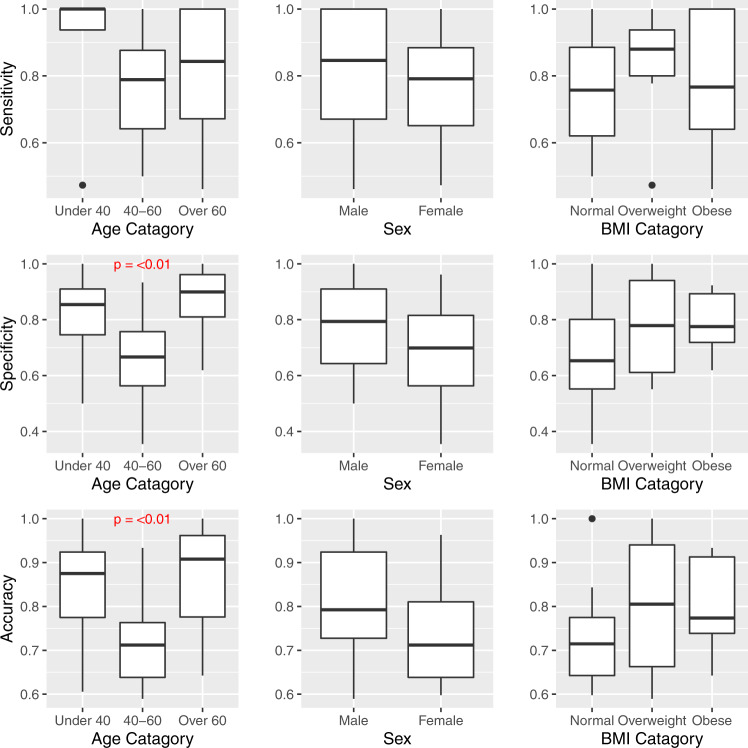
Model metrics stratified by demographic characteristics. Center line = median; upper bound of box = 75th percentile; lower bound of box = 25th percentile; and whiskers = minimum to maximum range, with extreme outliers denoted by black dots. *P* value based on Kruskal–Wallis *H* test. BMI Body Mass Index.

**Fig. 4:**
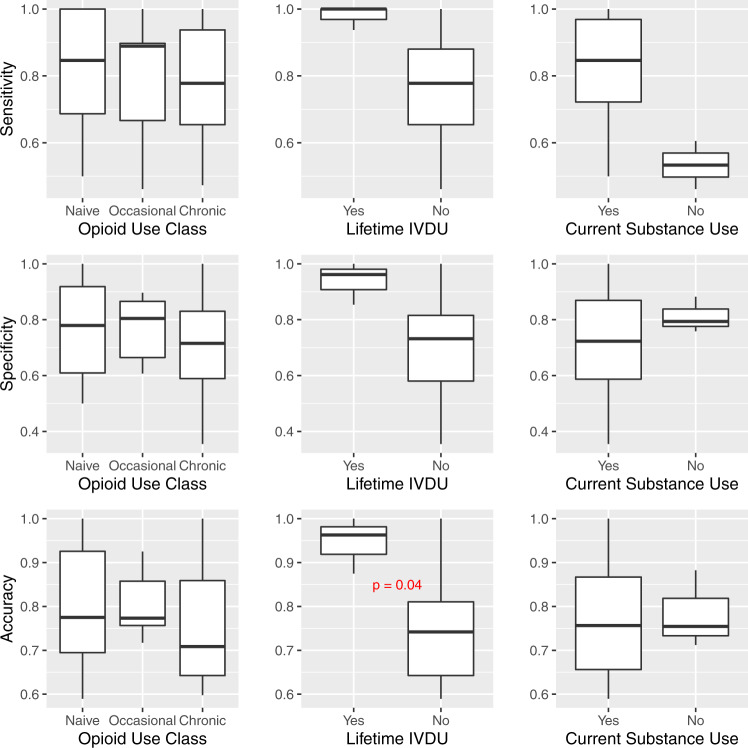
Model metrics stratified by substance use history. Center line = median; upper bound of box = 75th percentile; lower bound of box = 25th percentile; and whiskers = minimum to maximum range, with extreme outliers denoted by black dots. *P* value based on Wilcoxon rank-sum test. IVDU intravenous drug use.

Performance by select subgroups are displayed graphically in Figs. 3, 4. Graphical results for the remaining subgroups tested are presented in the Supplementary Material (Supplementary Figs. 1–3).

With respect to age, the model was most accurate and specific in the oldest and youngest groups of participants (those over 60 and under 40 years of age, respectively) compared to the middle age groups (40–60 years of age), and these differences were statistically significant (Fig. 3). In the small subset of participants with a history of intravenous drug use (IVDU), the model was more accurate compared to those without a history of IVDU (95% vs. 75%, respectively, *p* = 0.04, Fig. 4). With regard to opioid use classification, the negative predictive value of the model was significantly better in participants categorized as naive or those with occasional opioid use (compared to those with chronic use). Accuracy was also slightly higher in these groups, although not significantly (Fig. 4). No significant differences in model performance were found based on sex, body mass index (BMI), parity (for female participants), history of chronic pain, psychiatric history, lifetime history of tobacco use, lifetime history of alcohol use, or current substance use type. Due to lack of racial diversity in the sample, racial subgroups could not be evaluated.

### Model performance stratified by treatment characteristics

Treatment characteristics explored included predominant type of opioid administered, total MME administered over the study course, and duration of hospitalization. The impact of concomitantly administered medications from classes of particular interest (beta-adrenergic antagonists (or beta-blockers), calcium channel blockers (CCB), sedatives, stimulants, and non-narcotic analgesics) were also evaluated.

**Fig. 5:**
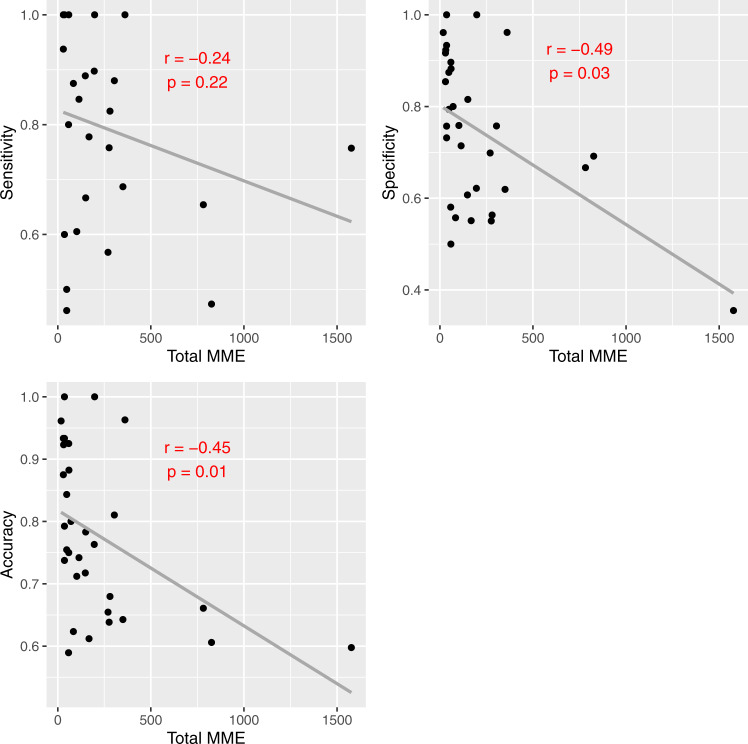
Model metrics vs. total MME administered. *R* and *P* values based on Pearson correlation. MME morphine milligram equivalents.

**Fig. 6:**
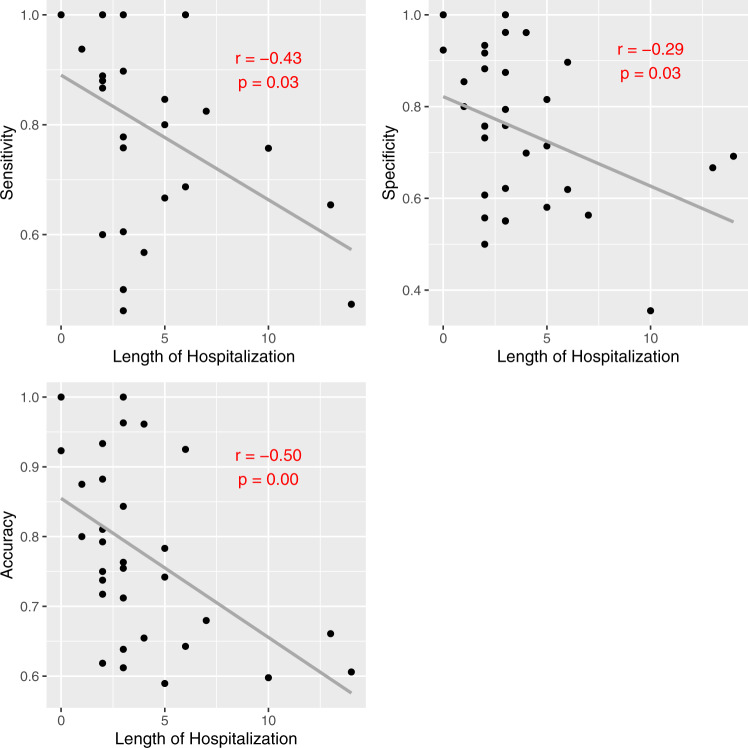
Model metrics vs. duration of hospitalization. *R* and *P* values based on Pearson correlation.

Model metrics were negatively correlated with both total MME administered (Fig. 5) and total duration of hospitalization (Fig. 6). Specificity and accuracy showed a significant downward trend as total MME increased. Similarly, sensitivity, specificity, and accuracy all decreased significantly as length of hospitalization increased. No significant differences were noted based on predominant opioid type administered.

**Fig. 7:**
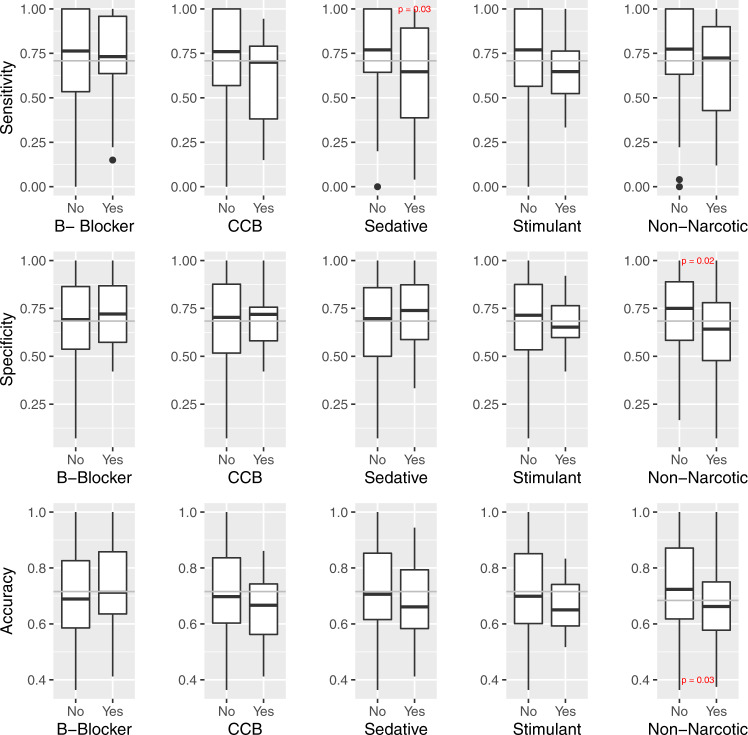
Model metrics by daily co-administered medication status. Center line = median; upper bound of box = 75th percentile; lower bound of box = 25th percentile; and whiskers = minimum to maximum range, with extreme outliers denoted by black dots. *P* value based on Wilcoxon rank-sum test. B-blocker beta-adrenergic antagonist, CCB calcium channel antagonist.

The impact of concomitantly administered medications from other classes was evaluated by considering opioid detection model metrics for each participant-day, stratified by whether a given class of medication was co-administered on that day. Model metrics by daily co-administered medication administration status are shown in Fig. 7. Accuracy and specificity were significantly decreased on study days where a non-narcotic analgesic was co-administered (74% vs. 66%, and 72% vs. 62%, respectively). Sensitivity decreased significantly on days when a sedative was co-administered (75.5% vs. 61.0%). Model metrics (sensitivity and accuracy) decreased on days where participants received a calcium channel blocker or stimulant in addition to opioid analgesics compared to those that did not; however, these differences in model performance were not statistically significant. There was no change in model performance based on beta-blocker co-administration.

## Discussion

In a sample of 36 hospitalized patients receiving repeated doses of intravenous opioids, as hypothesized, opioid administrations were detectable using a machine leaning model on physiologic wearable sensor data—an important innovation for the field. Several statistical features derived from the wearable sensor data changed notably from pre- to post-administration; specifically, accelerometry-based features decreased overall, while skin temperature, EDA, and HRV-based features increased overall. The final best performing model did not utilize these statistical features, however, but used raw sensor data in a format that considered both temporal and spatial data relationships to make decisions. It performed best in data windows with ample data (100 min) where the opioid administration occurred in the center of the window (i.e., the model had at least 30 min of data before and after the moment of administration to analyze). Overall, model performance was fair to good; but there is room for improvement prior to clinical deployment. Older (greater than 60 years) and younger (less than 40 years) age categories, and a history of IVDU were associated with significantly better model performance. Co-administration of non-narcotic analgesics, higher total MME administered, and longer duration of hospital stay were associated with significantly poorer model performance. Administration of cardioactive and sedative medications during the hospital stay were associated with a small decrease in model performance, although importantly, these decreases were not statistically significant in this sample.

Even though the temporal convolutional neural networks-based deep learning model performed better with raw data than with calculated statistical features, the calculated features do provide some insight about the physiologic phenomenon being captured in the sensor data surrounding opioid use. Significant changes in data were generally consistent with known opioid physiology and prior work^9^, which provides reassurance that the phenomenon being capturing is in fact opioid physiology. Decreases seen with accelerometry are expected due to general sedation and psychomotor slowing seen with opioids; in cases with mild opioid effect, individuals’ movements are slow and impaired, in more extreme cases (such as opioid toxicity), individuals are comatose and thus exhibit minimal limb movement. Increases in EDA and skin temperature shortly after opioid administration initially may seem counter-intuitive for a drug class whose effects are overall more sympatholytic in nature. But the relationship of opioids with the autonomic nervous system is complex, with evidence for opioid activation of both the parasympathetic and sympathetic nervous systems^20–23^. However, we expect that these changes are due to the well-established association between opioid use, histamine release, and concomitant vasodilation^24,25^, resulting in a brief period of warming/increase in conductance at the surface of the skin, as opposed to an increase in sympathetic nervous system activity. Increases in HRV parameters may be related to increased parasympathetic tone in a more relaxed physical state: this is consistent with, but not specific to, opioid effect.

These physiologic changes are expected to be sensitive but not specific for opioid use. So when relying on physiologic parameters as a measure of opioid detection, consideration must be given to alternative agents that impact the same physiology. Common agents expected to do this include sympatholytics (i.e., beta-blockers, calcium channel blockers), sedatives (i.e., benzodiazepines, barbiturates), and stimulants (i.e., amphetamines). Also, as analgesia may play some role in the observed physiology, other non-opioid analgesics (i.e., acetaminophen, non-steroidal anti-inflammatory drugs) may also impact changes. Exploring these variables in our dataset demonstrated that there seems to be a consistent decrease in model performance in individuals who receive concomitant medications (specifically on the days when these medications are co-administered), but these were largely not statistically significant decreases. Interestingly, the exception were non-narcotic analgesics, which were associated with a significant decrease in model performance. Although this needs to be explored in a larger dataset, these data provide confidence that our model can be expected to perform similarly in patients taking concomitant medications which act on the sympathetic nervous system, and that the contribution of pain (and analgesia) to our signal should be explored more carefully.

The negative correlation between model performance and both length of hospitalization and total MME administered was unexpected. In both cases, this may be related to a few outliers that had extreme values compared to the rest of the sample. An alternative hypothesis is that the participants who received more opioids were overall sicker, or had more pain. Consistent with previous work, there were differences based on opioid use history which we hypothesize are related to differences in physiologic adaptations (i.e., tolerance and dependence); however, using the present model, these were not as strong as previously noted. This will be further explored in future work.

One of the core challenges of mobile sensing in the space of OUD is that labeled data is generally limited. Any complex machine learning model trained on a small labeled dataset is susceptible to model bias. This paper aims to investigate the potential biases that our model might have across different demographic, historical and comorbidity related factors. The first step for potentially removing these biases from our model is to identify and recognize the biases which is the main objective of the paper. In the present dataset, our model performed slightly better for certain groups based on individual and treatment characteristics. From a computational standpoint, there are several steps that can be taken to compensate for these biases. If the training data is imbalanced with respect to a particular group/factor and consequently the model has seen significantly more data from a specific group/factor, the resulting model can be biased. One way to avoid the bias is to ensure uniform data collection across different groups and factors. Another approach to compensate for model bias is to augment the training data with synthetic data. Using prior knowledge about how different groups and factors behave with respect to the wearable signals, synthetic augmented datasets can be created that will emulate data collection from diverse groups and factors. A model that is trained with such a large synthetic augmented data can achieve a higher generalizability across different factors and groups. This is also a way to inject domain knowledge in the machine learning pipeline.

Our approach has several strengths and limitations, largely related to the generalizability of the findings to a more broad population. Although participants were hospitalized, this was not a completely controlled lab setting; they had some degree of freedom to conduct activities of daily living (e.g., walking in-patient rooms/halls, eating, drinking, etc.). We view this as a strength which supports the capability of the model to detect opioid administrations despite background noise of everyday life. However, multiple limitations also impact generalizability specifically, inclusion of patients with a single painful diagnosis (pancreatitis), and low racial and ethnic diversity. The inability to evaluate performance based on racial subgroups due to low N compounded this problem. Given recent insights into differences in wearable sensor data collection across skin tones^8,26–28^, the possibility that models may perform differently in non-Caucasian individuals should be explored. Another important limitation is the inability to account for the contribution of change in pain to the overall clinical picture. We attempted the collect electronic medical record (EMR)-reported pain scores pre- and post-opioid administration to include in the models; however, these were very poorly documented and there was not enough data to be usable. This will be an important parameter to collect prospectively for future work. Our relatively small sample size is a limitation, both from the diversity standpoint described above and from a computational standpoint. Larger datasets would allow more robust model evaluation on completely unseen test data, and will be necessary for future work. Finally, the current algorithm only considered intravenous opioid administration which is the least common route of administration in the outpatient setting (when considering therapeutic use). Pharmacokinetic differences (i.e., decreased bioavailability and delays due to absorption time) are expected to make changes associated with oral opioid ingestions more gradual in onset, and thus less physiologically pronounced. This may pose a challenge for the models, and will be addressed in future work.

Despite these limitations, this work advances the field towards the goal of creating an automated opioid detection system in several ways. First, it provides evidence that repeated opioid exposures can be detected in longitudinal data streams. The model is also able to detect not only if an opioid administration occurred in a 1 h window, but also when. Second, it provides insight into the physiologic changes being captured by the sensor data, providing some level of interpretability and explainability to the model. Notably, the physiologic signal changes are consistent with known opioid effect, adding confidence to this strategy. Finally, understanding the impact of participant-level and situational factors on the model accuracy provides insight on the expected limitations of the system in practice: i.e., which concomitantly administered drugs are expected to impact accuracy and in which patients the system will work best. It also provides information on which features should be included in future models and which are inconsequential. Future work in this space should be aimed at validating this model in- and out-of-hospital settings, with other routes of administration (particularly oral), and in diverse populations. Consideration should also be given to incorporating personal (i.e., age) and situational (i.e., co-administered medications) characteristics into a broadly applied model, or prospectively stratifying individuals based on categories of interest (i.e., drug use history, sex) and building unique models for subgroups to improve performance.

## Methods

### General study overview

**Fig. 8:**
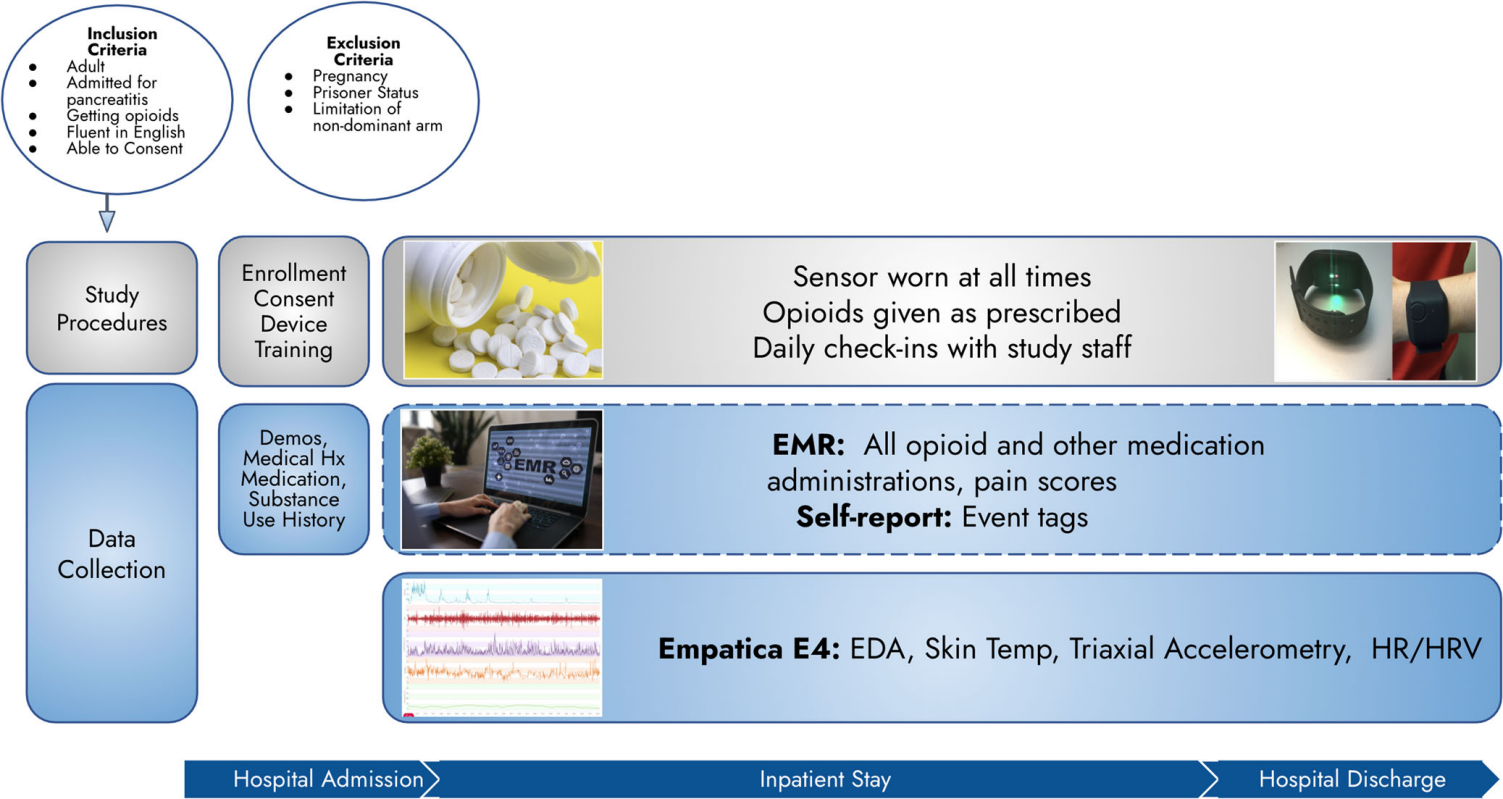
Flow diagram of study participation. BMI Body Mass Index, EMR Electronic Medical Record, EDA Electrodermal Activity, HR Heart Rate, HRV Heart Tare Variability.

All study-related procedures were reviewed and approved by the UMass Chan Medical School (UMass Chan) Institutional Review Board (IRB). This was an observational study of adult patients receiving opioid analgesics for an acute painful condition. Participants were asked to continuously wear a wrist-mounted sensor and record all opioid doses received while in the in-patient care setting (Fig. 8). Potential participants were identified through screening of the electronic Emergency Department (ED) tracking board at a large tertiary care academic medical center during normal business hours (9 a.m.–5 p.m.). Individuals who screened in were approached while in the ED, eligibility criteria were confirmed, and written informed consent was obtained. Enrollment, device training, and initial interviews were conducted in the participant’s hospital-based treatment room.

### Inclusion and exclusion criteria

To be included in the study, patients needed to: (1) be 18 years of age or older; (2) be admitted to the hospital for acute or chronic pancreatitis; (3) have a treatment plan which included pain management with opioid analgesics; (4) be fluent in English; and, (5) be capable of providing informed consent. Patients with pancreatitis were selected because this condition is generally managed in the in-patient setting with intravenous opioid analgesics due to its characteristic severe pain. Patients were excluded from the study if they were: (1) pregnant; (2) currently under police custody; or, (3) had an amputation or other significant limitation of motion (i.e., acute orthopedic injury) of the non-dominant arm that would preclude sensor wear.

### Wearable sensor data collection

**Fig. 9:**
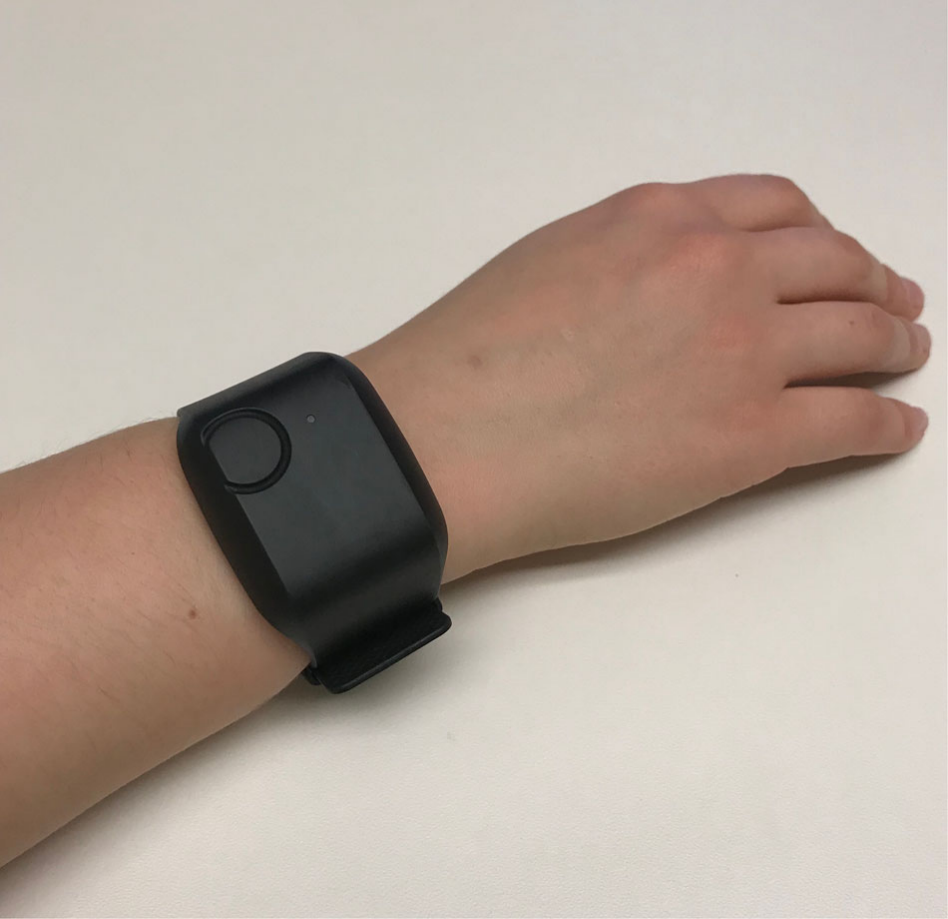
Empatica E4. Photo by author, device purchased by author.

A commercially available, noninvasive sensor (E4, Empatica Inc., Boston, MA, USA, Fig. 9) was used to collect physiologic study data. The research-grade device is water-resistant and has a battery life capable of recording continuously for 48 h on a single charge. Empatica employs a 128-bit data encryption strategy and does not record any direct identifiers on the device. The E4 continuously detects and records skin temperature (in degrees Celsius (C) at a rate of 4 Hz), triaxial accelerometry (in units g at a rate of 32 Hz), electrodermal activity (in microsiemens at a rate of 4 Hz), and heart rate/heart rate variability (measured via a photoplethysmography sensor at a rate of 64 Hz). All sensor data were stored in the device’s on-board integrated memory until downloaded to Empatica’s Health Insurance Portability and Accountability Act (HIPAA)-compliant cloud-based server (Empatica Connect) by research staff.

Participants wore the E4 on their non-dominant wrist from the time of study enrollment until hospital discharge and were instructed to press the event marker button on the device to indicate any opioid administration. Daily check-ins were conducted by research staff to exchange sensors with fully charged ones to ensure continuity of data acquisition and device functionality.

### Non-biometric data collection

#### Demographic and historical data

All non-biometric data was recorded and stored in the Research Electronic Data Capture (REDCap) data management platform^29^. Baseline information was collected on all participants including demographics, medical/psychiatric history, surgical history, and medication history (including home medications at the time of hospital admission). A detailed substance use history was also obtained, including an assessment of past and current opioid use (both licit and illicit). All baseline data was verified to the extent possible in participant’s electronic medical records, and any discrepancies between EMR data and self-report were reconciled during follow-up interviews. Throughout the study period, clinical data was abstracted from the EMR including route, dose, type, and timing of all medications administered, and prescriptions given at discharge. All opioid administrations were converted to morphine milligram equivalents^30^. Pre- and post-opioid administration pain scores were also abstracted from the EMR; however, the documentation of these data was notably inconsistent, and the degree of missingness precluded use in the final analysis.

#### Defining opioid use history classification

A spectrum of opioid use history types were considered that ranged from individuals who are opioid-naive, to those who use opioids chronically. Such distinctions are important to consider in the context of the physiologic adaptations (i.e., opioid tolerance and dependence). No standard definition exists to classify individuals on this spectrum, and existing ones used in the literature vary widely^31–33^. The classification definitions used were informed by prior literature and content expert consensus, and focused on identification of extreme outliers (i.e., those that most clearly fit into either extreme end of the spectrum) with many participants falling into the middle category (occasional opioid use). The opioid use history of each participant was classified independently by two study team members after review of all available self-report and EMR data related to past opioid use, using the following definitions:

##### Opioid-naive

No provider-prescribed opioids within the past 6 months, and no lifetime history of opioid misuse.

##### Chronic opioid use

Maintained on provider-prescribed opioids (i.e., for chronic pain) at the time of study enrollment, ongoing opioid misuse/Opioid Use Disorder (OUD), or a history of OUD with <5 years of abstinence.

##### Occasional opioid use

Not meeting criteria above for naive or chronic opioid use.

Any discrepancies that arose in classification were discussed by both reviewers until consensus was reached.

#### Defining opioid administration events

For all opioid administrations during the study period, there were two opportunities to capture the ground truth data; participant report (annotation of data via sensor event marker button press), and/or by clinical documentation in the EMR. For administrations where there was a participant-generated annotation and an EMR-documented administration time within 10 min (i.e., opioid use is simultaneously indicated between both sources of information), the participant annotation time was used as the ground truth opioid administration time. For instances where there was documentation of an opioid administration in the EMR without an associated annotation from the participant (assuming the participant forgot to annotate the event), the EMR recorded time was used as the ground truth opioid administration time. Only intravenous, discreet dose opioid administrations were used in this analysis; administrations via the oral, transdermal, and continuous infusion routes were excluded, as administrations with such significant pharmacokinetic differences (particularly in absorption and elimination) will require alternative modeling strategies.

### Sensor feature performance analysis

To understand how sensor-measured features behave surrounding an opioid administration (and to provide insight into the phenomena we are aiming to model), sensor data was compared 15 min pre-opioid administration and 30 min post-opioid administration. Both time and frequency-domain features were extracted from the available sensor data streams (Table 3), for a total of 51 features. Time-domain features indicate signal change over time. Frequency-domain features are complementary to time-domain features; they indicate how much a signal lies within each given frequency over a range of frequencies, and allow for observation of unique signal characteristics that cannot simply be observed in the time domain. Respective feature values were compared via students t-test to determine which demonstrated significant changes.

### Machine learning model development

Raw sensor data files were downloade from Empatica Connect in comma-separated values (CSV) format, and uploaded to Python^34^ for analysis. Data pre-processing included screening for invalid data (which may have resulted form improper device wear, poor connection with skin, etc.) and removing data points that were outside physiologic ranges (i.e., skin temperature < 20 degrees C, brief HR spikes > 200 beats per minute (bpm), and EDA values of zero).

The data was then split into 100 min segments with a sliding window length of 20 min, and characterized as either having an opioid administration occur within the window (positive class) or not (negative class). Machine learning models of varying complexity were used including logistic regression, Bidirectional Long Short-Term Memory (BiLSTM)^35^, Temporal Convolutional Network (TCN)^36^, Convolutional Neural Network Long Short-Term Memory (CNN-LSTM)^37^, Long Short-Term Memory with Fully Convolutional Network (LSTM-FCN)^38^, and CTA-TCN^39,40^. Models were trained using both raw sensor data and the calculated statistical features described above. The outcome was framed as both a classification problem (i.e., binary decision of whether or not an opioid administration occurred in the window of data tested) and as a regression problem (i.e., when in the data segment the opioid administration occurred). Leave one subject out cross validation (LOSOXV) was used for model testing. Briefly, in LOSOXV, data from one participant is withheld and all remaining data is used for training; then the model is subsequently tested on the withheld participant. This process is repeated N times (with *N* = number of participants) and the results are averaged. Classification models (for binary detection) were compared on sensitivity, specificity, weighted F-1 score, and area under the receive operator characteristic curve (AUC), and regression models (for opioid timing detection) were compared using mean-absolute error. The best performing model was selected based on these parameters.

### Statistical analysis of individual and treatment characteristics and impact on model performance

After selecting the best performing model, we sought to understand the relationship of individual and treatment characteristics to overall model performance (i.e., whether our model performed better in certain individuals or under certain treatment conditions). Overall model performance metrics (specificity, sensitivity, positive predictive value (PPV), and negative predictive value (NPV)) were calculated for each participant. To explore which factors impacted our model, we stratified participants into subgroups based on demographics: age, sex, race, BMI, historical data (opioid and other substance use history, psychiatric history, and chronic pain history), and treatment characteristics (type and amount of opioids received, duration of hospitalization, and co-administered medications). Model performance was then compared across subgroups. Descriptive statistics for baseline characteristics were calculated for the sample. Hypothesis testing was performed to compare model performance across categories: for normally distributed variables, students t-test (binary variables) and ANOVA (greater than two groups) were used, and for non-normally distributed variables, Wilcoxon rank sum (binary variables) and Kruskal–Wallis H (greater than two groups) were used. Simple correlations (Pearson’s r or Spearman’s r) were used to evaluate continuous variables. All statistical analyses was performed in R^41^.

### Reporting summary

Further information on research design is available in the Nature Research Reporting Summary linked to this article.

## Supplementary Information

Supplemental Material

Reporting Summary

## Data Availability

De-identified data will be made freely available, to qualified academic investigators for non-commercial research as required by the National Institutes of Health (NIH) Grants Policy on Sharing of Unique Research Resources and as permitted by the UMass Chan IRB. Investigators must submit a formal request for data to the Principal Investigator (stephanie.carreiro@umassmed.edu) who will grant permission to release the data as long as it meets the following requirements: (1) institution-specific permission to use the data for research, (2) guarantee that data will be used for research purposes only, and (3) completion of a standard data use agreement.

## Code availability

Code will be made freely available, to qualified academic investigators for non-commercial research. Investigators may submit a request for code to the Tauhidur Rahman (trahman@cs.umass.edu) who will grant permission to release the code.
