# Supplementary material for: Impact of individual and treatment characteristics on wearable sensor-based digital biomarkers of opioid use
Source: NPJ Digit Med. 2022 Aug 22;5:123. doi: 10.1038/s41746-022-00664-z (PMC9395337; doi:10.1038/s41746-022-00664-z)
Supplement: Supplementary file 1 — Supplemental Material [file 41746_2022_664_MOESM1_ESM.pdf]

Sensor data stream	Feature	F Value
Heart Rate	Maximum	2.72
	Mean	2.56
	SD	2.40
Skin Temperature	Maximum	-3.04
	Standard Deviation	-3.90
	Interquartile Range	-3.15
Electrodermal Activity	Minimum	3.26
	Skew	-4.90
	Kurtosis	-5.50
Accelerometry	Minimum	3.40
	Mean	3.00
	Mean Frequency	-3.50
	SD Frequncy	-3.06
Interbeat Interval	NN50	2.17
	pNN50	2.17
	SDNN	2.18
	VLF	-2.15
	LF	-2.66
	LF (nu)	-2.50

Supplementary Table 1: Features Calculated from Raw Sensor Data with Statistically Significant Changes from Pre- to Post-Opioid Administration

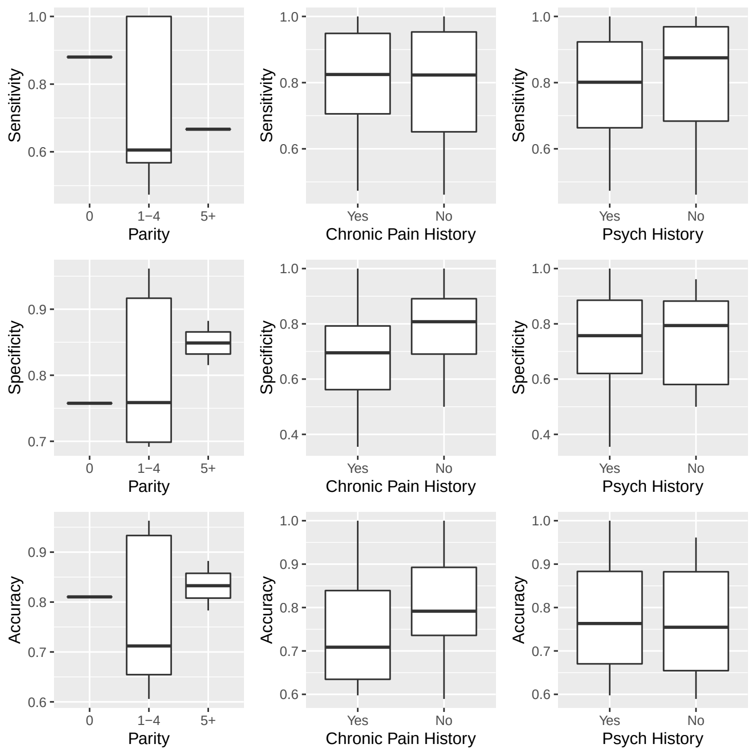

Supplementary Figure 1: Model Metrics Stratified by Additional Demographic Characteristics

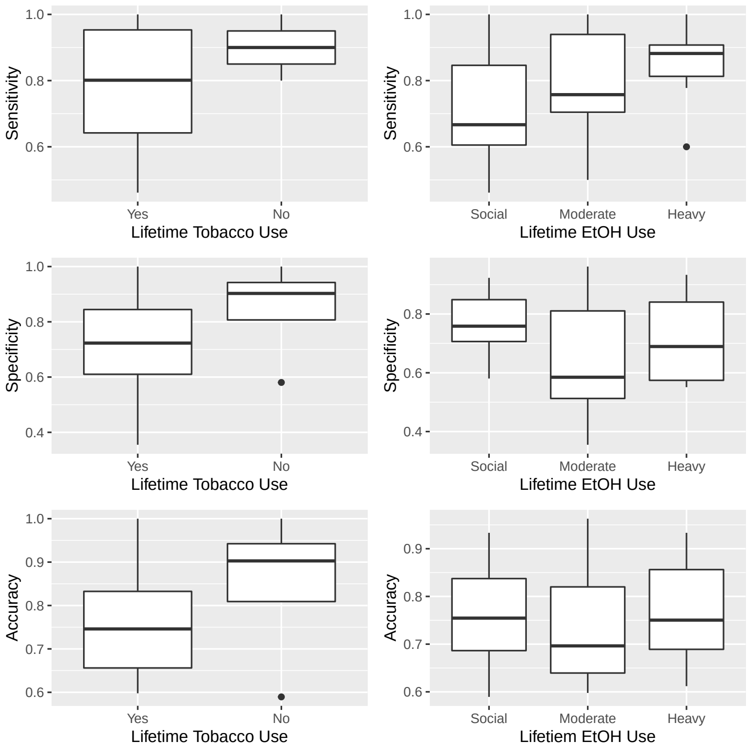

Supplementary Figure 2: Model Metrics Stratified by Lifetime EtOH and Tobacco Use

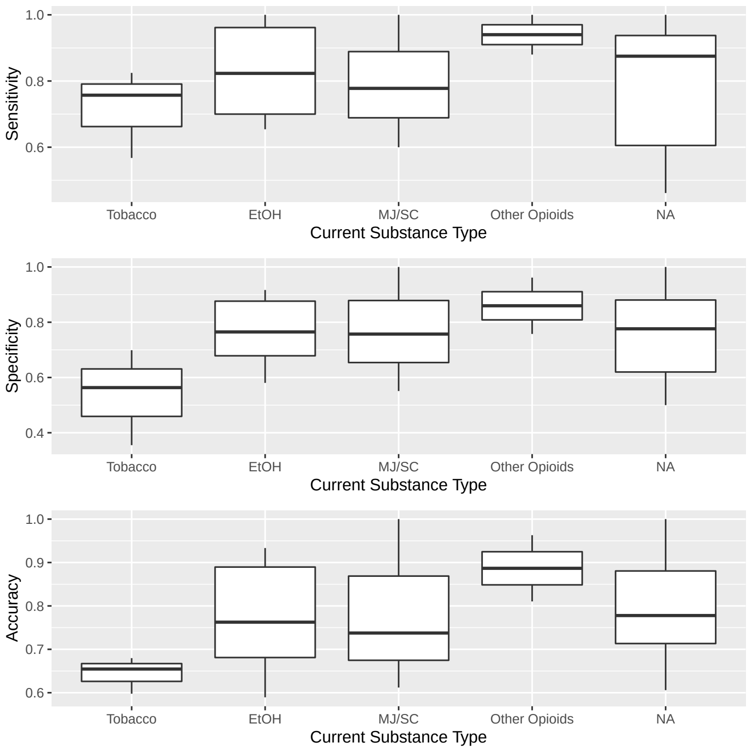

Supplementary Figure 3: Model Metrics Stratified by Current Substance Use Type
