# Supplementary material for: Impact of individual and treatment characteristics on wearable sensor-based digital biomarkers of opioid use
Source: NPJ Digit Med. 2022 Aug 22;5:123. doi: 10.1038/s41746-022-00664-z (PMC9395337; doi:10.1038/s41746-022-00664-z)
Supplement: Supplementary file 2 — Reporting Summary [file 41746_2022_664_MOESM2_ESM.pdf]

nature portfolio

Corresponding author(s): Stephanie Carreiro

Last updated by author(s): May 26, 2022

Reporting Summary

Nature Portfolio wishes to improve the reproducibility of the work that we publish. This form provides structure for consistency and transparency in reporting. For further information on Nature Portfolio policies, see our Editorial Policies and the Editorial Policy Checklist.

Statistics

For all statistical analyses, confirm that the following items are present in the figure legend, table legend, main text, or Methods section.

n/a	Confirmed	
☐	☒	The exact sample size (*n*) for each experimental group/condition, given as a discrete number and unit of measurement
☐	☒	A statement on whether measurements were taken from distinct samples or whether the same sample was measured repeatedly
☐	☒	The statistical test(s) used AND whether they are one- or two-sided<br>*Only common tests should be described solely by name; describe more complex techniques in the Methods section.*
☐	☒	A description of all covariates tested
☐	☒	A description of any assumptions or corrections, such as tests of normality and adjustment for multiple comparisons
☐	☒	A full description of the statistical parameters including central tendency (e.g. means) or other basic estimates (e.g. regression coefficient) AND variation (e.g. standard deviation) or associated estimates of uncertainty (e.g. confidence intervals)
☐	☒	For null hypothesis testing, the test statistic (e.g. *F*, *t*, *r*) with confidence intervals, effect sizes, degrees of freedom and *P* value noted<br>*Give P values as exact values whenever suitable.*
☒	☐	For Bayesian analysis, information on the choice of priors and Markov chain Monte Carlo settings
☐	☒	For hierarchical and complex designs, identification of the appropriate level for tests and full reporting of outcomes
☐	☒	Estimates of effect sizes (e.g. Cohen's *d*, Pearson's *r*), indicating how they were calculated

Our web collection on statistics for biologists contains articles on many of the points above.

Software and code

Policy information about availability of computer code

Data collection	Empatica Connect, RedCAP
Data analysis	Python 13.10.0, R 4.1.3

For manuscripts utilizing custom algorithms or software that are central to the research but not yet described in published literature, software must be made available to editors and reviewers. We strongly encourage code deposition in a community repository (e.g. GitHub). See the Nature Portfolio guidelines for submitting code & software for further information.

Data

Policy information about availability of data

All manuscripts must include a data availability statement. This statement should provide the following information, where applicable:

- Accession codes, unique identifiers, or web links for publicly available datasets
- A description of any restrictions on data availability
- For clinical datasets or third party data, please ensure that the statement adheres to our policy

De-identified data will be made freely available, to qualified academic investigators for non-commercial research as required by the National Institutes of Health (NIH) Grants Policy on Sharing of Unique Research Resources and as permitted by the UMass Chan IRB. Investigators must submit a formal request for data to the Principal Investigator (stephanie.carreiro@umassmed.edu) who will grant permission to release the data as long as it meets the following requirements: (1)

institution-specific permission to use the data for research, (2) guarantee that data will be used for research purposes only, and (3) completion of a standard data use agreement.

Human research participants

Policy information about studies involving human research participants and Sex and Gender in Research.

Reporting on sex and gender	Model performance was stratified by biologic sex. Sex and gender data were obtained from the electronic medical record, and were verbally verified with participants by research staff. Biologic sex was deemed the most appropriate variable for stratification (as opposed to gender identity because of the physiologic nature of the phenomenon being examined. Also of note, in our sample, all participants reported cisgender (i.e. biologic sex was identical to gender in all cases).
Population characteristics	Relevant covariates tested were: age, sex, race, BMI, historical data (opioid and other substance use history, psychiatric history, and chronic pain history), and treatment characteristics (type and amount of opioids received, duration of hospitalization, and co-administered medications)
Recruitment	Participants were identified by screening the electronic patient tracking board in the emergency department and through patients schedules for surgical clinics for patients who may meet inclusion criteria. Potential participants were approached by research staff, and offered the opportunity to participate.
Ethics oversight	University of Massachusetts Chan Medical School

Note that full information on the approval of the study protocol must also be provided in the manuscript.

Field-specific reporting

Please select the one below that is the best fit for your research. If you are not sure, read the appropriate sections before making your selection.

☒ Life sciences ☐ Behavioural & social sciences ☐ Ecological, evolutionary & environmental sciences

For a reference copy of the document with all sections, see nature.com/documents/nr-reporting-summary-flat.pdf

Life sciences study design

All studies must disclose on these points even when the disclosure is negative.

Sample size	The primary outcome of this study is an algorithm that uses wearable sensor data to predict opioid use. Unlike the design of clinical trials with a statistically measurable endpoint, adequate power to detect detection is conducted through repeat training within a dataset until there is acceptable agreement between the algorithm and ground truth. We enrolled a sufficient number of participants (N = 36) to collect 2070 hours of data and 339 discreet dose opioid administration events, which provided sufficient data for model training and testing. Our overall sample size is on the larger size for mHealth sensor-based studies in the development phase
Data exclusions	Only intravenous, discreet dose opioid administrations were used in this analysis; administrations via the oral, transdermal, and continuous infusion routes were excluded, as administrations with such significant pharmacokinetic differences (particularly in absorption and elimination) will require alternative modeling strategies.<br>Additionally, sensor data identified as invalid (non-consistent with physiologic data) in the pre-screening process were excluded. These data may have resulted form improper device wear, poor connection with skin, etc. Points outside physiologic ranges (i.e., skin temperature <20 degrees C, brief HR spikes > 200 beats per minute (bpm), and EDA values of zero were used as criteria to identify invalad data.
Replication	All details of the data collection and model building have been provided to ensure reproducibility.
Randomization	As this was an observational study, no randomization was conducted.
Blinding	As this was an observational study and the device is physically obvious, no blinding was conducted.

Reporting for specific materials, systems and methods

We require information from authors about some types of materials, experimental systems and methods used in many studies. Here, indicate whether each material, system or method listed is relevant to your study. If you are not sure if a list item applies to your research, read the appropriate section before selecting a response.

Materials & experimental systems

n/a	Involved in the study
☒	☐ Antibodies
☒	☐ Eukaryotic cell lines
☒	☐ Palaeontology and archaeology
☒	☐ Animals and other organisms
☒	☐ Clinical data
☒	☐ Dual use research of concern

Methods

n/a	Involved in the study
☒	☐ ChIP-seq
☒	☐ Flow cytometry
☒	☐ MRI-based neuroimaging
